# Supplementary material for: Leucyl-tRNA Synthetase Inhibitor, D-Norvaline, in Combination with Oxacillin, Is Effective against Methicillin-Resistant Staphylococcus aureus
Source: Antibiotics (Basel). 2022 May 18;11(5):683. doi: 10.3390/antibiotics11050683 (PMC9137938; doi:10.3390/antibiotics11050683)
Supplement: Supplementary file 1 [file antibiotics-11-00683-s001.zip › antibiotics-1699512-supplementary.pdf]

Supplementary data

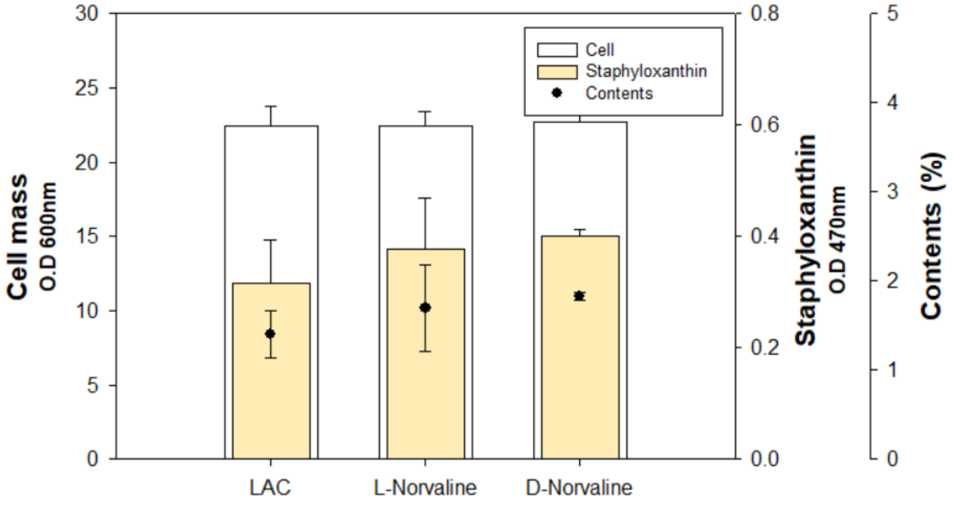

Supplementary Figure S1. Staphyloxanthin production in LAC and L- / D- Norvaline treated LAC.

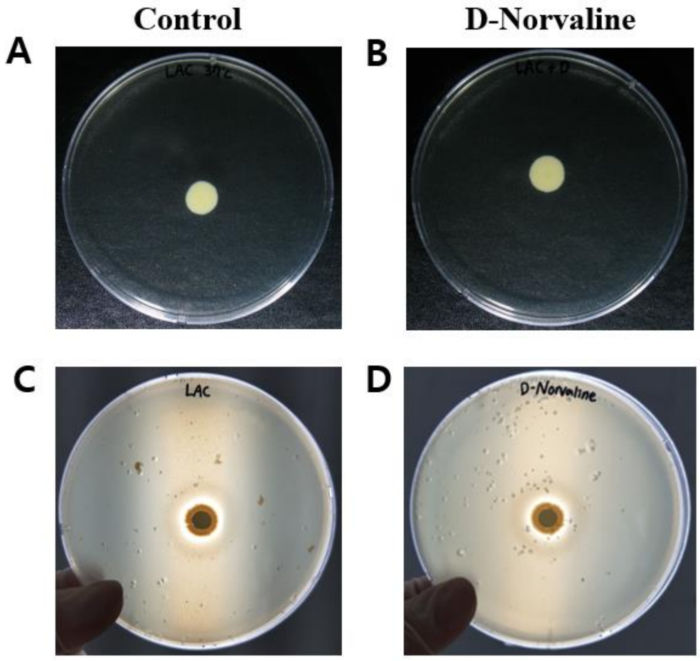

Supplementary Figure S2. Plate-based assay. (**A**) Motility of LAC. (**B**) Motility of LAC with D-Norvaline. (**C**) Protease activity in LAC. (**D**) Protease activity in LAC with D-Norvaline.

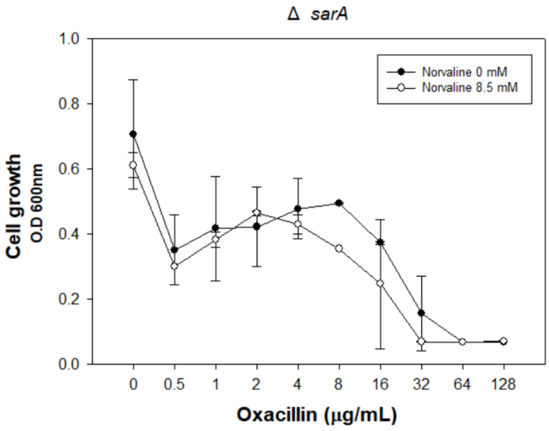
Δ sarA
Norvaline 0 mM
Norvaline 8.5 mM
Cell growth
O.D 600nm
0.0
0.2
0.4
0.6
0.8
1.0
0 0.5 1 2 4 8 16 32 64 128
Oxacillin (μg/mL)

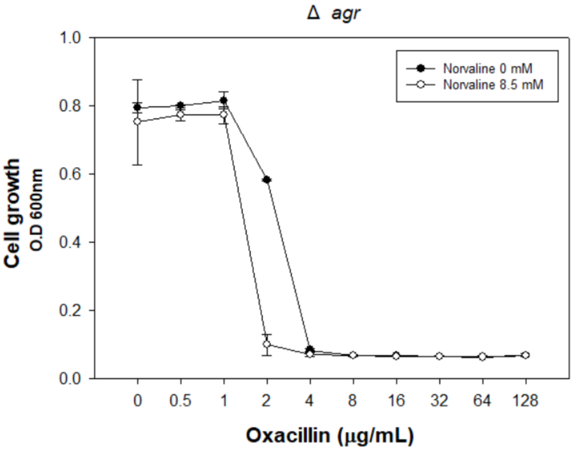
Δ agr
Norvaline 0 mM
Norvaline 8.5 mM
Cell growth
O.D 600nm
0.0
0.2
0.4
0.6
0.8
1.0
0 0.5 1 2 4 8 16 32 64 128
Oxacillin (μg/mL)

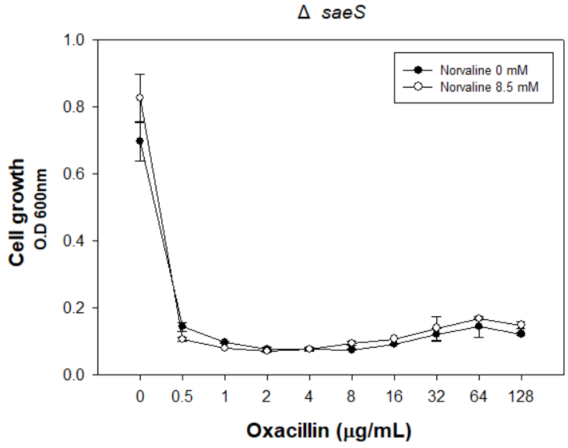
Δ saeS
Norvaline 0 mM
Norvaline 8.5 mM
Cell growth
O.D 600nm
0.0
0.2
0.4
0.6
0.8
1.0
0 0.5 1 2 4 8 16 32 64 128
Oxacillin (μg/mL)

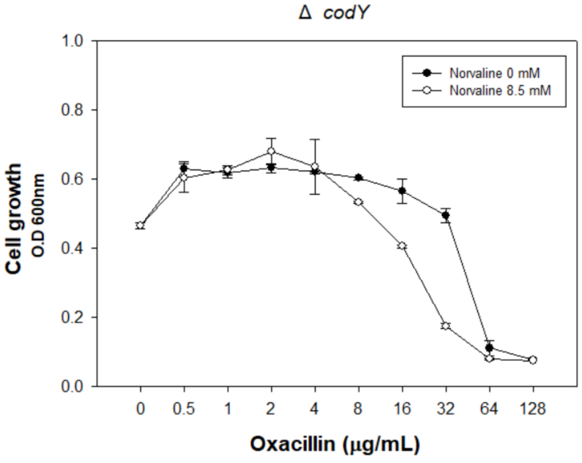
Δ codY
Norvaline 0 mM
Norvaline 8.5 mM
Cell growth
O.D 600nm
0.0
0.2
0.4
0.6
0.8
1.0
0 0.5 1 2 4 8 16 32 64 128
Oxacillin (μg/mL)

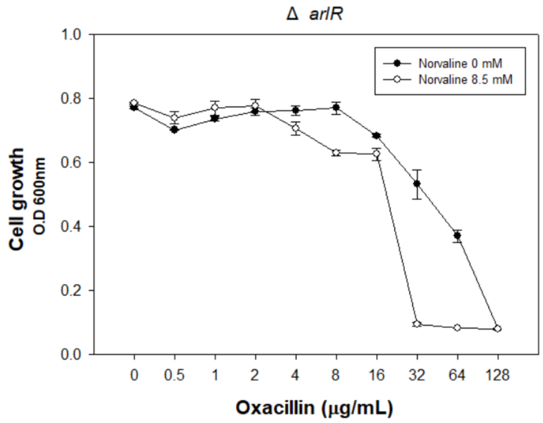
Δ arlR
Norvaline 0 mM
Norvaline 8.5 mM
Cell growth
O.D 600nm
0.0
0.2
0.4
0.6
0.8
1.0
0 0.5 1 2 4 8 16 32 64 128
Oxacillin (μg/mL)

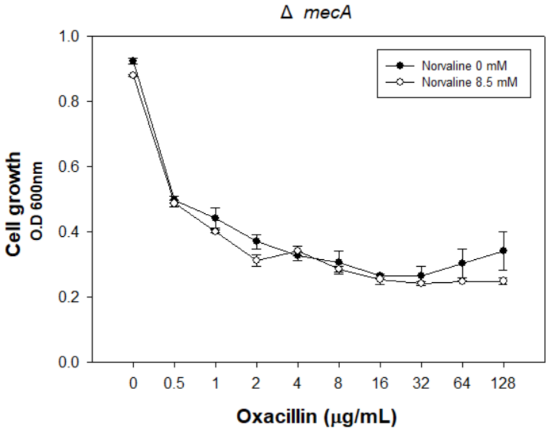
Δ mecA
Norvaline 0 mM
Norvaline 8.5 mM
Cell growth
O.D 600nm
0.0
0.2
0.4
0.6
0.8
1.0
0 0.5 1 2 4 8 16 32 64 128
Oxacillin (μg/mL)

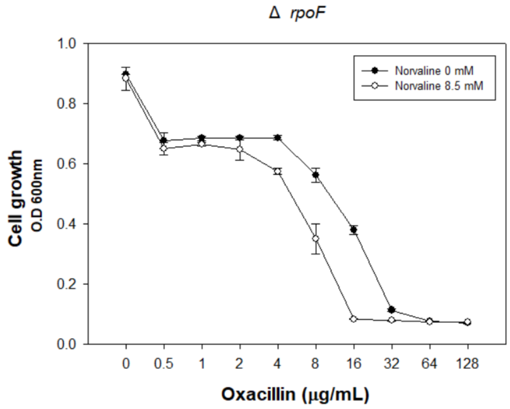
Δ rpoF
Norvaline 0 mM
Norvaline 8.5 mM
Cell growth
O.D 600nm
0.0
0.2
0.4
0.6
0.8
1.0
0 0.5 1 2 4 8 16 32 64 128
Oxacillin (μg/mL)

Supplementary Figure S3. Cell growth of MRSA deletion mutants (*ΔsarA, Δagr, ΔsaeS, ΔcodY, ΔarlR, ΔmecA,* and *ΔrpoF*) with and without D-norvaline treatment.
